# The Use of Protease Inhibitors in Pregnancy: Maternal and Fetal Considerations

**DOI:** 10.1155/2015/563727

**Published:** 2015-11-05

**Authors:** Elaine Duryea, Fiona Nicholson, Sara Cooper, Scott Roberts, Vanessa Rogers, Donald McIntire, Jeanne Sheffield, Robert Stewart

**Affiliations:** Department of Obstetrics and Gynecology, The University of Texas Southwestern Medical Center, Dallas, TX 75390, USA

## Abstract

*Background*. Previous studies examining protease inhibitor use in pregnancy and the rate of preterm and small-for-gestational-age infants have yielded conflicting results.* Methods*. This was a retrospective study of HIV-infected women who delivered singleton infants at our institution between 1984 and 2014. Women with protease inhibitor use were compared to women on regimens without a protease inhibitor as well as those who received no antepartum antiretroviral therapy. Infants were considered preterm if less than 37 completed weeks of gestation and small-for-gestational-age if less than 10th percentile.* Results*. During the study period 1,004 pregnancies met inclusion criteria. Of those, 597 received a protease inhibitor as part of their regimen, 230 ART without a protease inhibitor, and 177 no ART. There was no difference in the rate of preterm birth between groups who received ART with or without a protease inhibitor, 14% versus 13%. There was no difference in the rate of small-for-gestational-age infants between the three groups. Use of a protease inhibitor was associated with a greater fall in viral load during pregnancy, *p* < 0.001.* Conclusion*. In this population with access to prenatal care and ART, treatment with protease inhibitors was associated with a greater fall in viral load, but not an increase in small or preterm infants.

## 1. Introduction

The rate of new HIV infections in the United States has remained stable over recent years, with an annual infection rate in women of 9,500 cases [1]. Despite this, HIV remains a significant public health concern, with 1.1 million HIV-infected people currently living in the United States. In addition, HIV-infected women are more frequently becoming pregnant and present a unique challenge to clinicians. Antiretroviral therapy, recommended for all pregnant women, serves two major functions. The first is treatment of maternal HIV infection to prevent progression of disease. The second is to provide chemoprophylaxis to prevent vertical transmission to the fetus. Antiretroviral therapy decreases the risk of vertical transmission through reduction of maternal viral load as well as transplacental transfer of the drug for preexposure prophylaxis. Guidelines published by the National Institutes of Health (NIH) clearly state that all pregnant women should receive combined antiretroviral therapy regardless of disease status. However, selection of the optimal treatment regimen is less straightforward [2].

Combination antiretroviral therapy, consisting of two nucleoside reverse transcriptase inhibitors (NRTIs) plus either a nonnucleoside reverse transcriptase inhibitor (NNRTI) or a protease inhibitor, is recommended for all HIV-infected pregnant women. The recommendations do acknowledge that while there is associated risk, the benefits of maternal treatment for both maternal and fetal health outweigh these risks. Each class of antiretroviral drug has a unique set of possible side effects. For example, NRTIs have been associated with mitochondrial toxicity in rare individuals, while nevirapine (a NNRTI) has been associated with hepatotoxicity and rash, and efavirenz (also a NNRTI) has been associated with fetal neural tube defects with early first trimester exposure [2–6]. Protease inhibitor use in pregnancy has been inconsistently associated with both preterm birth and small-for-gestational-age infants, with a possible etiology of decreased progesterone levels recently postulated [7–13].

All possible effects of combination antiretroviral therapy on the pregnancy must be taken into consideration when counseling women with HIV infection and providing recommendations for therapy. The objective of this study was to determine whether protease inhibitor use in our population is associated with preterm birth or small-for-gestational-age infants. Our null hypothesis is that there is no difference in the rate of preterm birth or small-for-gestational-age infants in women receiving a protease inhibitor as part of their prescribed treatment regimen.

## 2. Materials and Methods

This was a retrospective cohort study of HIV-infected women who delivered a singleton live born infant at our institution from January 1984 through April 2014. This study was approved by the Institutional Review Board of the University of Texas Southwestern Medical Center and Parkland Hospital. All HIV-infected women who delivered at our institution during the study period were identified and their medical records were reviewed for demographic information, markers of HIV disease status, class of antiretroviral therapy, and delivery information.

Over the study period the treatment of HIV infection in pregnancy evolved, and consequently the treatment provided at our institution changed along with national treatment guidelines. Prior to 1990 no therapy was available, followed by a period of time when women received either single or multiple nucleoside reverse transcriptase inhibitors. In 1997 combination ART with a protease inhibitor became first-line at our institution and was usually well tolerated. There were only two indications for a woman to be on an alternate regimen: either she was well controlled at presentation for prenatal care on an acceptable regimen, or she had a direct contraindication to the preferred treatment regimen.

Women with a protease inhibitor as part of the prescribed treatment regimen were compared to those on ART without a protease inhibitor as well as to women who received no antepartum ART. Demographic information and markers of HIV disease status such as duration of diagnosis, CD4 cell count, and HIV viral load were examined for association with treatment received. Delivery outcomes including birth weight and gestational age were then examined to evaluate differences in the rate of preterm birth or small-for-gestational-age infants. Infants were identified as premature if born at less than 37 completed weeks of gestation and small-for-gestational-age if they were less than the 10th percentile for gestational age based upon an updated national reference [14]. Logistic regression adjusting for ethnicity, age, duration of diagnosis, CD4 count at initiation of prenatal care and delivery, and HIV viral load at start of prenatal care and delivery (as linear effects) was then performed. Statistical analyses included Pearson chi-square, Student's *t*-test, Wilcoxon rank sum, and logistic regression, with *p* values less than 0.05 considered significant. Analysis was performed using SAS 9.2 (SAS Institute Inc., Cary, NC).

## 3. Results

During the study period 1,004 singleton live births were delivered to 792 HIV-infected women who had medication usage, gestational age at delivery, and birth weight available for analysis. Of those pregnancies, 597 (59.5%) received a protease inhibitor as part of their treatment regimen, 230 (22.9%) received ART without a protease inhibitor, and 177 (17.6%) received no antepartum ART. Of note, 144 of the 177 women who did not receive antepartum ART delivered their infants after 1990, when ART was available at our institution. These women were either diagnosed at the time of delivery, or unable to demonstrate compliance with recommended ART and therefore represent a fundamentally different group of women. As shown in Table 1, ethnicity was not different between the three groups. Women who did not receive antepartum ART were more likely to be nulliparous than women who received some form of therapy (*p* = 0.005), while women who received a protease inhibitor were older than those that did not (*p* < 0.001). When examining women who received prenatal care, women who did not receive antepartum ART presented later in gestation, a median of 23.5 weeks, compared to women who received treatment both with or without a protease inhibitor, median of 12 and 13.5 weeks, respectively, *p* < 0.001.

When examining markers of maternal disease status, the duration of diagnosis at delivery was significantly shorter, a median of less than 3 months, for women who did not receive antepartum ART as compared to the other two groups (Table 2). This is a result of the number of women who were diagnosed at the time of delivery. Additionally, women who received a protease inhibitor had been diagnosed with HIV for a longer duration of time at presentation for prenatal care than women who received ART without a protease inhibitor (median 2.0 years versus 1.0 year, *p* < 0.001).

**Figure 1:**
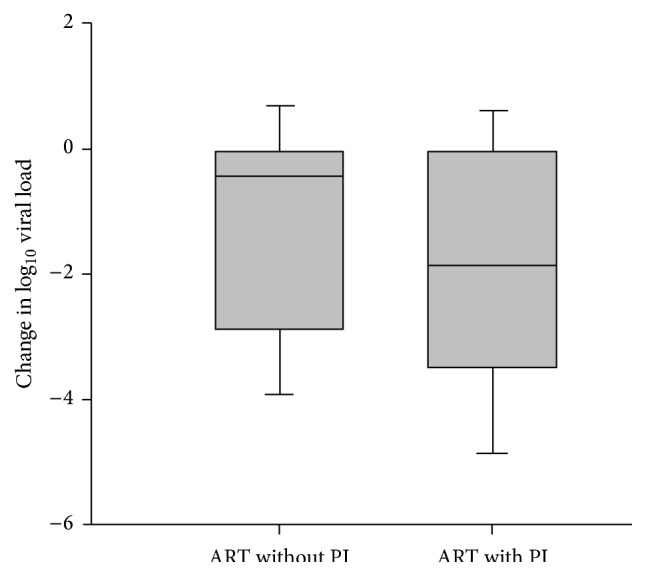
Change in viral load during pregnancy for women receiving ART with and without a protease inhibitor. Shaded area represents one standard deviation from the mean, which is denoted as the black line. Whiskers represent two standard deviations from the mean. The change in log⁡10 viral load is significantly greater for ART with a protease inhibitor (PI) using the Student-Newman-Keuls test, *p* < 0.001.

HIV viral load and CD4 cell count at both presentation for prenatal care and delivery were then examined. CD4 cell count at presentation for prenatal care was higher in women who did not receive ART when compared to those who received some form of antepartum ART with a median of 557 cells/mm^3^ (*p* = 0.001) and did not change significantly between presentation and delivery. In comparison, CD4 cell counts at presentation for prenatal care for women receiving antepartum ART both with and without a protease inhibitor improved significantly over the duration of pregnancy but did not differ between the groups with median values of 463 and 456 cells/mm^3^ at presentation and 524 and 505 cells/mm^3^ at delivery, respectively. Viral load at presentation for prenatal care was higher in women who did not receive antepartum ART, median 4,770 copies/mL, compared to women who received some form of antepartum ART. The median viral load for women who received no ART fell slightly by the time of delivery, median 2,540 copies/mL, despite a lack of regular ART therapy in this group. Of note, when comparing those women who received ART with and without a protease inhibitor, viral load at presentation for prenatal care was higher in women receiving a protease inhibitor, median values of 2,054 and 1,372 copies/mL, respectively. However, this discrepancy reversed by the time of delivery, as illustrated in Figure 1, with viral load at delivery significantly lower in women receiving a protease inhibitor, *p* < 0.001.

There was a significant difference in gestational age at delivery between the three groups as shown in Table 3, *p* = 0.003, though the absolute difference was small. There was no difference in the rate of premature birth between the two groups of women who received ART with and without a protease inhibitor, with rates of 14% and 13%, respectively. However, women with no antepartum ART had a 21% rate of preterm birth, which was significantly higher compared to the other two groups, *p* = 0.049. This trend continued with examination of preterm birth less than 34 weeks of gestation, with similar rates of 4 and 5% for the groups of women who received ART with and without protease inhibitors and a much higher rate of 10% for those women who did not receive antepartum ART, *p* = 0.01. When examining birth weight, infants born to women who did not receive antepartum ART weighed less, mean 2,904 grams, as compared to those infants born to women receiving ART with and without a protease inhibitor, mean of 3,080 and 3,010 grams, respectively, *p* = 0.02. However, when adjusted for gestational age, the rate of small-for-gestational-age infants did not differ between the three groups.

Logistic regression was then performed to adjust for ethnicity, maternal age, and duration of diagnosis, as well as viral load and CD4 cell count at both presentation for prenatal care and delivery as shown in Table 4. When comparing women who received no antepartum ART to those women who received ART with a protease inhibitor, the unadjusted odds ratio for preterm birth was significantly elevated at 1.7 with CI (1.1, 2.6); however the adjusted odds ratio following regression analysis was 1.0. The odds ratio for preterm birth both prior to and following regression analysis was not different between the two groups of women who received some form of ART. As previously shown in Table 3, unadjusted risks for small-for-gestational-age infants did not vary between the groups, which did not change following logistic regression.

## 4. Discussion

In our population, use of protease inhibitors as part of antiretroviral therapy in pregnancy was not associated with preterm birth or small-for-gestational-age neonates. HIV infection itself has been associated with both preterm birth and low birth weight infants, though these rates have shown some decrease over time [15]. Specifically, the national preterm birth rate for the United States is approximately 12% [16]; however the reported rate among HIV-infected women is often higher, reported between 12 and 36% [7, 10, 11]. Therefore, while the rate of preterm birth in our study was higher than the national rate for HIV-uninfected women, it is comparable to the previously documented preterm birth rates in HIV-infected women.

Importantly, though women with protease inhibitor use in pregnancy presented to prenatal care with a higher viral load than those women who received ART without a protease inhibitor, by delivery this relationship reversed, reflecting the ability of protease inhibitors to rapidly and effectively minimize viral load. This may have direct clinical implications. Currently, all women with a viral load greater than 1,000 copies/mL at the time of delivery should be offered cesarean delivery as well as intrapartum zidovudine prophylaxis. In addition, maternal viral load at delivery is inversely related to the risk of vertical transmission [2]. Use of protease inhibitors in pregnancy may therefore decrease the need for cesarean delivery and intrapartum zidovudine and minimize vertical transmission to the infant. The data from our study strengthens the recommendation for the inclusion of protease inhibitors in the treatment regimen for HIV in pregnancy as preterm birth and small-for-gestational age infants were not increased with their use.

This is a large study of HIV-infected pregnant women at a single institution who received care by a specialized team of providers, a considerable strength. However, the retrospective nature of this study, which was conducted over a long study period, predisposes it to several confounding variables. Unfortunately, the majority of studies reviewing the risk of protease inhibitors have been retrospective, with only randomized trial conducted in the setting of the developing nation of Botswana [11]. In that study antiretroviral therapy was initiated late in pregnancy (at 26–34 weeks of gestation), and protease inhibitor use was associated with a preterm birth rate of 21% compared to 12% of women receiving the alternative regimen. In an attempt to control for the biases introduced in a retrospective study, we included analyses to control for HIV disease status at presentation for prenatal care and at delivery as well as ethnicity, and the results remained unchanged. However, information on alcohol and tobacco use and history of prior preterm birth was not available for the entire cohort, all of which have been associated with small-for-gestational-age infants and/or preterm birth [17–19]. Despite these limitations, we are confident that in our population protease inhibitor therapy does not appear to be associated with poor fetal growth or preterm birth.

Protease inhibitors are usually well tolerated in pregnancy and have been widely adopted for the treatment of women with HIV both during and outside of pregnancy. This study provides reassurance to clinicians that protease inhibitors are not associated with preterm birth or small-for-gestational-age infants in a population with early access to antiretroviral therapy and regular prenatal care. At this time, the optimal regimen for the treatment of pregnant women with HIV infection in the United States is not clear, and the NIH currently lists two treatment regimens as first-line options: either a protease inhibitor or a NNRTI, plus two NRTIs. Unfortunately, NNRTIs have been associated with rapid development of resistance, limiting future treatment options [20]. At this time, our study provides evidence of the benefit of protease inhibitor use in pregnancy and provides reassurance in regard to the risk of preterm birth or small-for-gestational-age infants.

## Figures and Tables

**Table 1:** Population characteristics in HIV-infected pregnant women by type of antepartum antiretroviral therapy.

	ART with a protease inhibitor	ART without a protease inhibitor	No ART	*p* value
	*n* = 597	*n* = 230	*n* = 177	
Race/ethnicity				0.5
Black	413 (69)	154 (67)	114 (64)	
Hispanic	112 (19)	43 (19)	33 (19)	
White	68 (11)	33 (14)	28 (16)	
Other	4 (1)	0 (0)	2 (1)	
Nulliparity	183 (31)	75 (33)	79 (45)	0.005
Age at delivery, years	27.5 ± 5.9	25.7 ± 7.4	24.7 ± 6.3	<0.001
Gestational age at presentation for prenatal care, weeks	12 [8, 21]	13.5 [9, 22]	23.5 [13, 34]	<0.001

Data reported as mean ± SD, median [quartiles], or *n* (%) as appropriate.

**Table 2:** Markers of maternal disease status during pregnancy in HIV-infected women by type of antepartum antiretroviral therapy.

	ART with a protease inhibitor	ART without a protease inhibitor	No ART	*p* value
	*n* = 597	*n* = 230	*n* = 177	
Duration of diagnosis, years	2.0 [0.5, 4.7]	1.0 [0.4, 2.7]	0.2 [0, 1.0]	<0.001
Viral load, copies/mL				
At presentation	2,054 [37, 20,965]	1,372 [0, 12,266]	4,770 [643, 20,400]	0.02
At delivery	0 [0, 251]	0 [0, 1347]	2,540 [0, 25,940]	<0.001
CD4 count, cells/mm^3^				
At presentation	463 [299, 626]	456 [314, 624]	557 [375, 747]	0.001
At delivery	524 [372, 693]	505 [349, 677]	565 [337, 717]	0.349

Data reported as median [quartiles].

**Table 3:** Selected pregnancy outcomes in HIV-infected women according to protease inhibitor use.

	ART with a protease inhibitor	ART without a protease inhibitor	No ART	*p* value
	*n* = 597	*n* = 230	*n* = 177	
Gestational age at delivery, weeks	38.0 ± 2.3	38.5 ± 2.2	37.8 ± 3.0	0.003
Preterm birth <37 weeks	82 (14)	31 (13)	37 (21)	0.049^*∗*^
Preterm birth <34 weeks	26 (4)	11 (5)	18 (10)	0.01^*∗*^
Birth weight, grams	3010 ± 607	3080 ± 657	2904 ± 686	0.02
SGA	116 (19)	54 (23)	39 (22)	0.4

Data reported as mean ± SD, median (quartiles), or *n* (%) as appropriate.

SGA: small-for-gestational-age defined as less than 10th percentile for gestational age.

^*∗*^ *p* values demonstrate a significance between women who did not receive ART and the other two groups.

**Table 4:** Unadjusted and adjusted odds ratios for pregnancy outcomes in HIV-infected women according to type of antepartum antiretroviral therapy.

	Unadjusted odds ratio	Adjusted odds ratio
Preterm birth <37 weeks		
ART without protease inhibitor	1.0 (0.6, 1.5)	0.9 (0.5, 1.5)
No ART	1.7 (1.1, 2.6)	1.0 (0.5, 2.0)
SGA^*∗*^		
ART without protease inhibitor	1.3 (0.9, 1.8)	1.3 (0.8, 1.9)
No ART	1.2 (0.8, 1.8)	1.1 (0.6, 2.0)

Logistic regression was performed to adjust for ethnicity, age, and duration of diagnosis, as well as viral load and CD4 cell count at both presentation for care and delivery.

Data reported as odds ratio (95% confidence interval) for women who received the indicated therapy as compared to women receiving a protease inhibitor.

^*∗*^SGA: small-for-gestational-age, defined as less than 10th percentile for gestational age.
